# Enhanced Functional Connectivity between the Bilateral Primary Motor Cortices after Acupuncture at Yanglingquan (GB34) in Right-Hemispheric Subcortical Stroke Patients: A Resting-State fMRI Study

**DOI:** 10.3389/fnhum.2017.00178

**Published:** 2017-04-10

**Authors:** Yanzhe Ning, Kuangshi Li, Caihong Fu, Yi Ren, Yong Zhang, Hongwei Liu, Fangyuan Cui, Yihuai Zou

**Affiliations:** ^1^Department of Neurology and Stroke Center, Dongzhimen Hospital, The First Affiliated Hospital of Beijing University of Chinese MedicineBeijing, China; ^2^Department of Internal Medicine, Gulou Hospital of Traditional Chinese Medicine of BeijingBeijing, China; ^3^The Key Laboratory of Internal Medicine of TCM, Ministry of Education, Beijing University of Chinese MedicineBeijing, China

**Keywords:** functional magnetic resonance imaging, primary motor cortex, acupuncture, subcortical stroke

## Abstract

Increasing neuroimaging researches in stroke rehabilitation had revealed the neural mechanisms of rehabilitation therapy. However, little was known about the neural mechanisms of acupuncture therapy in subcortical stroke patients. The aim of this study was to investigate the changes of functional connectivity (FC) between the bilateral primary motor cortices (M1s) after acupuncture intervention in right subcortical stroke patients. Twenty right-hemispheric subcortical stroke patients and 20 healthy subjects were recruited to undergo one functional magnetic resonance imaging (fMRI) scanning. The scanning consisted of resting-state fMRI before and after needling at Yanglinquan (GB34), and task-evoked fMRI. The most significant active point during the left passive thumb-to-index task was chosen as the seed point. The seed-based FC analysis of the bilateral M1s was performed. Stroke patients revealed decreased FC between the bilateral M1s compared with healthy subjects, and the decreased FC was significantly enhanced after acupuncture at GB34. Acupuncture could increase the intrinsically decreased FC between the bilateral M1s which provided further insight into the neural mechanisms of acupuncture for motor function recovery in stroke patients.

## Introduction

Stroke has been ranked as the leading cause of motor disability among adults across the world, which had brought heavy burden to the family and the society (Lozano et al., 2012). Motor impairments of limbs gravely affect their ability to perform activities of daily living (ADL), as well as social participation. The ability to live independently after stroke depends largely on the recovery of motor function. A large number of studies had demonstrated that adequate rehabilitation therapies could promote motor function recovery (Klamroth-Marganska et al., 2014; Liu et al., 2014; Saunders et al., 2014).

In recent years, functional magnetic resonance imaging (fMRI) has been introduced as a novel method to explore the reorganization of function and structure after stroke. The primary motor cortex (M1) is a brain region related with voluntary movement, which involves in motor function recovery. Abundant cross-sectional and longitudinal neuroimaging studies in subcortical stroke patients had confirmed that functional reorganization in the ipsilesional M1 existed (Pelicioni et al., 2016), and the resting-state functional connectivity (rsFC) between the bilateral M1s initially decreased and then it gradually increased during motor function recovery (Wang et al., 2010; Rehme et al., 2011; Zhang J. et al., 2014).

The efficacy of acupuncture on stroke rehabilitation were confirmed by numbers of randomized, controlled clinical trials (Kjendahl et al., 1997; Wayne et al., 2005; Zhang et al., 2015). Abundant reviews also indicated that acupuncture was beneficial for the post-stroke rehabilitation (Wu et al., 2010; Lim et al., 2015). According to the traditional Chinese medicine (TCM) theory, GB34, called Yanglingquan, was not only the “he” (meeting) point of the Gallbladder Meridian of Foot-Shao yang, but also was the influential point of tendons. GB34 was frequently chose in recovering motor function for stroke hemiplegia patients in clinical practice and trials (Fang et al., 2016; Ratmansky et al., 2016; Yang et al., 2016). A previous study on task related fMRI had revealed that needling at GB34 could induce some motor related brain regions overlapped key regions of the sensorimotor network (SMN; Na et al., 2009). Our previous studies also had confirmed that needling at GB34 in hemiplegic patients could increase motor-cognition connectivity as well as decrease contralesional compensation of M1 and enhance the (FC) of the default mode network (DMN; Zhang Y. et al., 2014; Chen et al., 2015). However, little is known about the changes of FC between the bilateral M1s with respect to acupuncture therapy after subcortical stroke.

Therefore, in the current study, we recruited 20 patients with right-hemispheric subcortical stroke and 20 healthy subjects as controls, and obtained task-evoked activation and rsFC between the bilateral M1s data via fMRI scanning. FC analysis was used to estimate FC between the bilateral M1s before and after needling at GB34 in both patients and controls. We postulated that (1) following from previous studies, the patients would show abnormal FC between the bilateral M1s compared to the healthy subjects. (2) The abnormal FC will be changed after needling at GB34, while no change occurs in healthy subjects. In this study, we only recruited ischemic stroke patients with subcortical infarctions involving the motor pathways. In order to eliminate the dominant effect of the left hemisphere, patients with right-handed before stroke and right hemispheric lesions were included.

## Materials and Methods

This study was approved by the First Affiliated Hospital of Beijing University of Chinese Medicine Ethics Committee. All subjects gave informed consent prior to inclusion in this study. This study was carried out in accordance with the recommendations of the Declaration of Helsinki.

### Participants

Twenty right-handed patients (13 males, aged 61.32 ± 8.53 years) were employed with left motor hemiparesis due to ischemic infarct of the corticospinal tract on the right hemisphere. The inclusion criteria were as follows: first-ever ischemic stroke; aged 35–75 years; subcortical lesion restricted to the right internal capsule (IC), basal ganglia (BG), corona radiate (CR), and its neighboring regions; within 6 months after the onset; without cognitive deficit. The exclusion criteria were as follows: recurrent stroke; any brain abnormalities; any other disease that may influence participation; any MRI contraindications. Most of enrolled patients were similar with our published article (Zhang et al., 2016). Another 20 healthy subjects (10 males, aged 58.86 ± 8.53 years) were recruited with no history of neurologic or psychiatric disorders.

### MRI Acquisition

Images were acquired using a 3.0 Tesla MRI scanner (Siemens, Sonata Germany) at Dongzhimen Hospital, Beijing, China. Prior to scanning, all participants were asked to rest for 20 min and were instructed to stay still, think of nothing in particular, keep eyes closed, and not to fall asleep during scanning. Earplugs were worn to attenuate scanner noise and foam head holders were immobilized to minimize head movements during scanning.

Prior to the functional scanning, high-resolution structural information for anatomical localization was acquired using 3D MRI sequences. Resting-state fMRI data was collected using a single-shot, gradient-recalled echo-planar imaging sequence with the following parameters: repetition time = 2000 ms, echo time = 30 ms, flip angle = 90°, matrix = 64 × 64, field of view = 225 mm × 225 mm, slice thickness = 3.5 mm, gap = 1 mm, 32 interleaved axial slices, and 180 volumes. The same parameters to resting-state scanning were used in the task-evoked fMRI with exception that 100 volumes were acquired.

### Experimental Paradigm

In the current research, we employed the non-repeated event-related-fMRI (NRER-fMRI) design to investigate the prolonged effects after needling, which was used in our previous study (Zhang Y. et al., 2014). Following the design paradigm of our previous study, we employed 1-min needle manipulation before 1-min resting epoch and followed by 8-min resting scan (without acupuncture manipulation). Acupuncture was performed at Yanglingquan (GB34, located in the lateral aspect of the posterior knee) on the left leg. Acupuncture was performed by inserting a sterile, single-use silver needle (25 mm in length and 0.30 mm in diameter) vertically into GB34 to a depth of 2–3 cm, and the stimulation included rotating the needle clockwise and counterclockwise at 1 Hz with even reinforcing and reducing manipulation for 60 s. All acupuncture procedures were performed by one licensed and skilled acupuncturist. Another 6-min 16-s resting scan was performed before the acupuncture procedure as the baseline. Eighteen patients and 20 controls were performed by needling at GB34.

De-qi is believed to be crucial to the therapeutic effectiveness of acupuncture (Bai et al., 2013). After each fMRI scanning, subjects were asked to fill questionnaires to record their experience of De-qi. As the sharp pain was thought to be an inadvertent noxious stimulation, we would rule out the subjects if they reported the sharp pain. Among all the subjects, no one reported the sharp pain.

### Task Design

All subjects performed a left passive thumb-to-index task with a frequency of 1 Hz. Cycles consisting of a 20 s resting block (baseline) followed by a 20 s left hand-grasping block were repeated five times. Patients with stroke performed the motor task using the left hand, whereas healthy controls used their left hand. Potential mirror movements of the unaffected hand were monitored carefully by the experimenters. No patient with observable mirror movements was excluded from the activation analysis.

### Image Processing

The resting-state data processing and analyzing were mainly carried out with the statistical parametric mapping software (SPM8^1^). A total of 174 volumes for each subject were corrected for slice timing after the starting volumes were discarded for signal equilibrium. The following steps were spatial realignment for head motions, normalization into the Montreal Neurological Institute (MNI) template, resampling into 2 × 2 × 2 mm^3^ voxels, smoothing with a Gaussian kernel of 4 × 4 × 4 mm^3^ full width at half-maximum, and linear regression. Two patients (subject 04 and subject 18) were ruled out for resting-state analysis, for exhibiting head motion >3 mm maximum translation and/or 3° rotation during the course of needling scans.

Similar processing steps were applied to the task-evoked data by using SPM8 software.

### Statistical Analysis

All the 40 subjects’ task-evoked data from the first level of General Linear Model (GLM) analysis were processed by One-sample *T*-test (two-tailed, *p* < 0.05, corrected by Monte Carlo Simulations, iterated 1000 times, and cluster size >94 voxels). The most significant active point during the left hand movement which located in the right precentral gyrus (MNI coordinate = 39, −33, 63) were extracted as the seed point for FC analysis. To perform the FC analysis, the seed region was extracted from the region of interest (ROI) centered within the range of a radius of 10 mm. The FC between the seed region and the rest of the brain was calculated with Fisher’s Z-transformation by using rest software^2^. The *Z* value images entered into *T*-test analyses. Two-sample *T*-test using SPM8 software (two-tailed, *p* < 0.05, corrected by Monte Carlo Simulations, iterated 1000 times, and cluster size >22 voxels) was conducted within the left M1 mask (M1 was extracted by using spm anatomy) between two groups. Paired *T*-test using SPM8 software (two-tailed, *p* < 0.05, corrected by Monte Carlo Simulations, iterated 1000 times, and cluster size >22 voxels) was conducted within the left M1 mask before and after needling at GB34 in both patients and controls. The reported statistics were color-coded and mapped in MNI space.

## Results

### Demographic and Clinical Information

**Figure 1:**
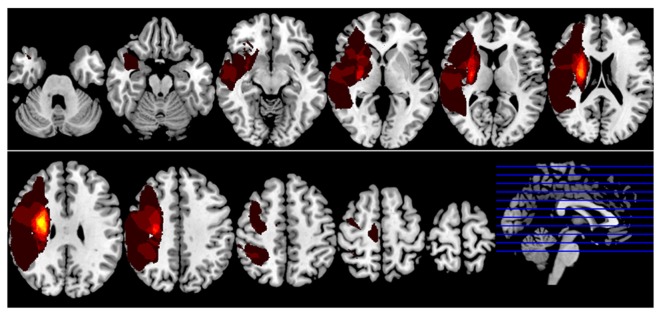
**Lesion incidence map of right hemispheric subcortical stroke patients**. All masks of stroke lesions were overlaid on T1 template in MNI standard space. The warm color represents a larger number of subjects in which voxels lay within a stroke lesion.

The basic demographic and clinical data were shown in Table 1. The results showed that all participants were right-hemispheric subcortical stroke patients with National Institute of Health Stroke Scale (NHISS) from 3 to 14 (mean value, 5.50 ± 3.12), Fugl-Meyer Assessment-upper limbs (FMA-U; mean value, 33.20 ± 18.71) and Fugl-Meyer Assessment-lower limbs (FMA-L; mean value, 23.30 ± 7.94). Compared with healthy subjects, stroke patients showed no significant differences in age (*p* = 0.286) and sex (*p* = 0.337). The duration from stroke onset to the MRI scan ranged from 18 to 161 days (mean value, 51.65 ± 36.77 days). The stroke lesions involved the BG, CR, IC and periventricular regions (PV). A stroke lesion map was plotted in Figure 1.

**Table 1:** **The basic demographic and clinical data of right-hemispheric subcortical stroke patients**.

Case	Gender	Age (years)	Handed	Lesion location	Days after stroke	NHISS	FMA-U	FMA-L
01	M	37	R	R, BG	20	7	25	22
02	M	58	R	R, BG	90	7	23	20
03	F	74	R	R, BG, PV	41	6	21	19
04	F	68	R	R, PV	24	4	56	28
05	M	48	R	R, BG, PV	29	4	19	31
06	F	66	R	R, PV, CR	20	3	53	34
07	M	63	R	R, CR, CS	44	4	18	32
08	F	55	R	R, BG, PV, CS	50	3	65	34
09	F	57	R	R, BG	34	8	21	22
10	M	57	R	R, BG	68	9	16	10
11	M	67	R	R, BG, CR	53	7	11	9
12	M	68	R	R, CS	73	5	50	30
13	M	65	R	R, BG, PV, CS	18	3	32	24
14	M	62	R	R, IC	23	3	56	23
15	M	59	R	R, BG, PV	107	3	33	25
16	F	66	R	R, BG, PV	18	14	10	9
17	M	71	R	R, BG	28	3	40	23
18	M	60	R	R, BG	161	3	56	32
19	M	61	R	R, CR, PV	48	11	6	15
20	M	57	R	R, BG	84	3	53	24

*BG, basal ganglia; CR, corona radiate; CS, centrum semiovale; F, female; FMA-U, Fugl-Meyer Assessment-upper limbs; FMA-L, Fugl-Meyer Assessment-lower limbs; IC, internal capsule; M, male; NIHSS, National Institute of Health Stroke Scale; PV, periventricular regions; R, right*.

### Task Activation Results

**Figure 2:**
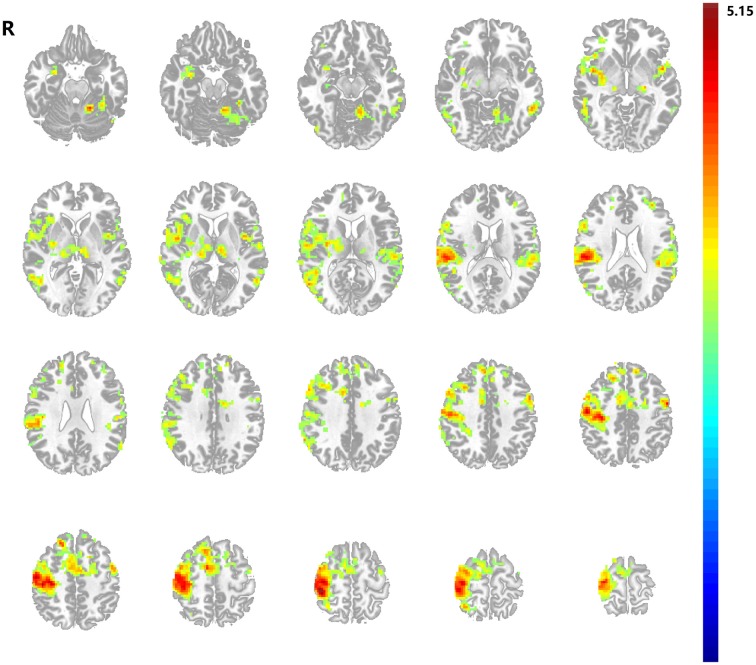
**Motor task activation map**. Stroke patients and healthy subjects revealed significant higher activations in a series of brain regions following left hand motor task. Results from two-tailed, *p* < 0.05, corrected by Monte Carlo Simulations, iterated 1000 times, and cluster size >94 voxels.

**Figure 3:**
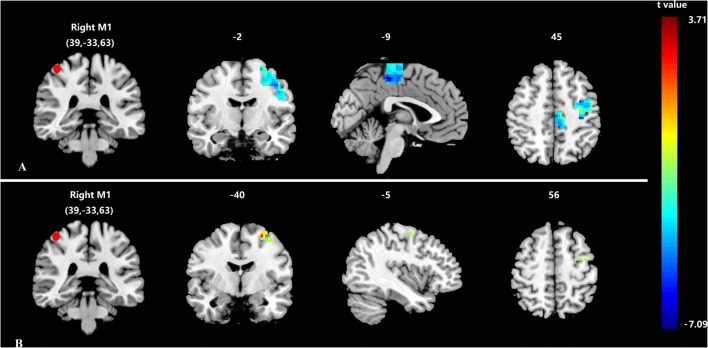
**Differences in functional connectivity (FC) of the bilateral primary motor cortices (M1s). (A)** Decreased FC of the bilateral M1s between stroke patients and healthy subjects; **(B)** Increased FC of the bilateral M1s between after and before acupuncture stimulation in stroke patients. Results from two-tailed, *p* < 0.05, corrected by Monte Carlo Simulations, iterated 1000 times, and cluster size >22 voxels. The red sphere represents the right M1 region of interest.

To detect the seed point for FC analysis, left passive thumb-to-index task was performed both in stroke patients and healthy subjects. During the task state, the motor task activated the right superior temporal gyrus, right middle frontal gyrus, bilateral insular, bilateral postcentral gyrus, bilateral inferior parietal lobule, bilateral middle temporal gyrus, bilateral thalamus and multiple motor related brain regions including the precentral gyrus and the secondary motor areas of both hemispheres in all participants (shown in Figure 2). The most significant active point which located in the right precentral gyrus (MNI coordinate = 39, −33, 63) was defined. The right M1 region of interest was shown in Figure 3.

### Functional Connectivity Results

To display temporal correlations between spatially remote neurophysiological processes, the ROI-based rsFC analysis was performed between bilateral M1s.

Before needling at GB34, compared with healthy subjects the stroke patients showed significantly decreased FC between the right M1 and left M1 (*p* < 0.05, corrected by Monte Carlo Simulations, iterated 1000 times, and cluster size >22 voxels, shown in Figure 3A). The peak MNI coordinate was (−3, −33, 54) and the peak *t* score was −7.0946.

After needling at GB34, the stroke patients showed significantly increased FC between the right M1 and left M1 in comparison with baseline (*p* < 0.05, corrected by Monte Carlo Simulations, iterated 1000 times, and cluster size >22 voxels, shown in Figure 3B). The peak MNI coordinate was (−27, −6, 60) and the peak *t* score was 3.2028. In addition, the healthy subjects showed no change between the bilateral M1s before and after acupuncture at GB34.

## Discussion

In the current study, we tried to investigate the neural mechanisms of acupuncture for motor function recovery in stroke patients. Our results indicated that acupuncture at GB34 could enhance the intrinsically decreased FC between the bilateral M1s in unilateral subcortical stroke patients.

A large number of studies had demonstrated the decreased FC between the bilateral M1s in patients following subcortical stroke (Grefkes et al., 2008; Park et al., 2011; Li Y. et al., 2016; Zheng et al., 2016), which were in line with our findings. Patients with subcortical stroke showed abnormalities in both function and structure (Zhang et al., 2016). The altered FC between the bilateral M1s could be interpreted by white matter damages in both the affected corticospinal tract and the transcallosal tracts following subcortical stroke (Lindenberg et al., 2010, 2012; Li Y. et al., 2015).

In this study, we fixed our attention on the treatment effect of acupuncture in subcortical stroke patients with motor deficit. We found significantly increased FC between the bilateral M1s in stroke patients after needling at GB34, while no change in healthy subjects.

Several previous studies had revealed that the enhanced M1-M1 rsFC played a beneficial role during the process of motor function recovery in stroke patients (Wang et al., 2010; Golestani et al., 2013; Liu et al., 2015). One recent study revealed that stroke patients with good outcomes of motor function recovery showed increased FC between the bilateral M1s (Liu et al., 2015). Moreover, the FC between the bilateral M1s was positively correlated with the degree of motor function recovery (Wang et al., 2010). One research from Li Y. et al. (2016), who also performed the rsFC analysis of stroke patients after 1-month antiplatelet treatment, showed that the enhanced FC between the bilateral M1s after treatment following the motor function recovery and daily living abilities. Another two recent studies on the change of FC between the bilateral M1s after 4-week rehabilitation intervention in chronic stroke patients exhibited the same result (Fan et al., 2015; Zheng et al., 2016). Based on these previous studies, we can see that the adequate rehabilitation treatment could accelerate the recovery process in stroke patients, and the enhanced M1-M1 rsFC had been closely associated with motor function recovery (van Meer et al., 2012; Yin et al., 2012).

Acupuncture as a main method of complementary therapies, is known to be effective to pain, neurodegenerative diseases, mood disorders, and so on Wang et al. (2011), Ashford et al. (2015), Chung et al. (2015). Numerous neuroimaging studies had demonstrated that acupuncture played a therapeutic role in knee osteoarthritis, migraine, mood disorders, by enhancing FC of related brain regions or networks (Chen X. et al., 2014; Li K. et al., 2015; Hwang et al., 2016; Li Z. et al., 2016). Accordingly, the enhanced FC between the bilateral M1s could also be interpreted as the motor function recovery in stroke patients.

Based on the theory of TCM, acupoints were functionally related to some global function such as skeletal muscle movement and played a vital role in different diseases with corresponding acupuncture treatment protocols (Yan et al., 2005). GB34 as the influential point of tendons was an important acupoint for functional modulation of muscles and tendons (Tsui and Leung, 2002). A previous study had revealed that needling at GB34 could modulate the cortical activities of somatomotor areas in healthy subjects (Jeun et al., 2005). Another study in healthy subjects by Zhang et al. (2004) showed that stimulation of GB34 inhibited bilateral M1s, and premotor cortex (PMC) by using task-state fMRI. For patients with Parkinson’s disease, needling at GB34 could activate the precentral gyrus and prefrontal cortex (Yeo et al., 2014). For stroke patients, it had been confirmed that acupuncture played an important role in improving regional cerebral blood flow (Lee et al., 2003). Moreover, acupuncture at Waiguan (TE5) could modulate the SMN of the bilateral hemispheres and increase cooperation of bilateral SMNs in stroke patients (Chen J. et al., 2014). Our previous studies also demonstrated that acupuncture at GB34 could modulate the rsFC within the DMN, and showed positive interaction effect in ipsilesional motor-related cortices and negative interaction effect in contralesional motor cortex (Zhang Y. et al., 2014; Chen et al., 2015). Hence, our results of the current study provided new evidence that acupuncture could accelerate the neural plasticity of motor function by enhancing the rsFC of the bilateral M1s after subcortical stroke.

This study might have some limitations. First, although we had limited the disease duration within 6 months, the range of duration were broad. Further studies with similar disease duration would be needed to confirm our results. Second, as in this study we only recruited right-hemispheric subcortical patients, the effect of acupuncture in left-hemispheric subcortical patients was unclear. The study on the change of M1-M1 rsFC after acupuncture intervention in left subcortical stroke patients will be needed in the future.

## Conclusion

Our study demonstrated that acupuncture at GB34 could enhance the decreased FC between the bilateral M1s in right-hemispheric subcortical stroke patients, which may reflect the mechanism of neural plasticity for acupuncture therapy.

## Author Contributions

YN conducted the experiments, analyzed the data, interpreted the results and statistical analysis and drafted the manuscript. KL analyzed the data, interpreted the results and statistical analysis, and drafted the manuscript. CF, HL, YZhang and YR conducted the experiments and assisted in interpreting the results. YZou and FC contributed to the study conception and study design and interpreted the results. All authors have read and approved of the manuscript.

## Conflict of Interest Statement

The authors declare that the research was conducted in the absence of any commercial or financial relationships that could be construed as a potential conflict of interest.

## Footnotes

^1^http://www.fil.ion.ucl.ac.uk/spm

^2^http://www.restfmri.net
